# Physiological and molecular mechanisms governing the effect of virus-free chewing cane seedlings on yield and quality

**DOI:** 10.1038/s41598-020-67344-4

**Published:** 2020-06-25

**Authors:** Kai-li Wang, Quan-qing Deng, Jian-wen Chen, Wan-kuan Shen

**Affiliations:** 1 College of Agriculture, South China Agricultural University, Guangzhou, 510642 China; 2 Scientific Observing and Experimental Station of Crop Cultivation in South China, Ministry of Agriculture, Guangzhou, 510642 China

**Keywords:** Plant physiology, Biotic

## Abstract

The effects of increasing yield and quality of virus-free chewing cane seedlings and their physiological and molecular basis were studied in this study. Results showed that compared with infected seedlings (the control), the yield of chewing cane stems grown from virus-free seedlings increased by 21.81–29.93%, stem length increased by 28.66–34.49 cm, internode length increased by 2.16–2.68 cm, the single stem weight increased by 20.10–27.68%, the reducing sugar increased by 0.91–1.15% (absolute value), and sucrose increased by − 0.06–1.33% (absolute value). The decrease in sucrose content did not reach significant level, but all other parameters were reached significant level. The chlorophyll content, photosynthetic parameters such as stomatal conductance (Gs), net photosynthetic rate (Pn) and transpiration rate (Tr), the activity of photosynthetic key enzymes ribulose-1,5-bisphosphate carboxylase (Rubisco) and phosphoenolpyruvate carboxylase (PEPC), and gene (*pepc*, *rbcS,* and *rbcL*) expression levels were all greater in virus-free seedlings than infected seedlings. The content of superoxide anion (O_2_^−^) and malondialdehyde (MDA) in virus-free seedlings was lower than infected seedlings at the main growth stage. With increased development, the activities of the antioxidant enzymes superoxide dismutase (SOD), peroxidase (POD), and catalase (CAT) were gradually higher in virus-free seedlings than infected seedlings. Our results indicate that virus-free seedlings may improve photosynthesis efficiency and promote photosynthesis by increasing chlorophyll content, photosynthetic key enzyme activity, and the gene expression levels in leaves. By increasing the activity of antioxidant enzymes, reducing the degree of membrane lipid peroxidation, and improving the stress resistance of chewing cane, the virus-free chewing cane seedlings increased yield and quality. Our findings provide a scientific and theoretical basis for the promotion and application of virus-free chewing cane seedlings.

## Introduction

*Saccharum* cultivar is a high photosynthetic efficiency C_4_ crop composed of both sugar cane (*Saccharum* hybrids spp.) and chewing cane (*Saccharum officinarum* L.) that is widely grown in tropical and subtropical regions. Sugar cane is a hybrid species that contains a high fiber and sucrose content, and is primarily grown for raw material for sugar production. Chewing cane is grown of its low fiber content and high reducing sugar content. It is also rich in a number of essential amino acids and iron elements and is primarily consumed as a fresh fruit^1^. Chewing cane is widely planted in many countries around the world, but especially in Southeast Asia, the South Pacific, and China^2^. In China, the planting area of chewing cane reached 230,000 hm^2^ per year, and output exceeded 35 million tons. Chewing cane is an asexually propagated crop (using seed cane as propagator), and the most widely grown cultivar is ‘Badila’ and similar variant lines^3^. After several decades of continuous planting, it has been found that chewing cane can become infected with the viruses: *Sugarcane mosaic virus* (SCMV), *Sorghum mosaic virus* (SrMV), *Sugarcane streak mosaic virus* (SCSMV), *Sugarcane bacilliform virus* (SCBV), and *Sugarcane yellow leaf virus* (SCYLV), as well as other viruses. After which, serious problems can arise, including degeneration, reduction in yield, and deterioration of quality. Thus, the presence of viruses can negatively influencing the economic benefits of production^1,4^.

Using virus-free seedlings is the primary way to control plant virus diseases^5,6^. Dewant et al.^7^ developed somatic embryo propagation technology to produce virus-free sugarcane seedlings, and were able to achieve a virus removal rate of 100% using growing medium containing Triazole Nucleoside (20 mg/L) and acyclovir (40 mg/L). A procedure for the in vitro elimination of SCMV, SrMV, SCSMV, and SCYLV from infected sugarcane has also been developed, and virus detection results found that the virus elimination rate following apical meristem culture was 61–92%, whereas the virus removal rate using axillary bud meristem culture was 90%^8^. In addition, SCSMV-free sugarcane was generated from infected plants using in vitro meristem tip culture technology^9^.

Virus-free seedlings tend to have greater yield and quality. The biomass and sucrose content of virus-free sugarcane seedlings has been significantly greater than those of common seedlings^2^. Compared with virus-free sugarcane seedlings, sugarcane plants with virus symptoms had a decreased internode length and fresh weight, but not a lower sucrose concentration^10^. Compared with common seedlings, virus-free seedlings resulted in an average of 16.75% higher stem yield, and a sucrose content that was 0.6% higher (absolute value)^11^. Wu et al.^12^ have also found that virus-free seedlings resulting from hot water treatment had a 9.5–14.7% greater stem yield and 0.68–1.69% greater sucrose content (absolute value).

Most previous work has focused on virus-free technologies and the effect on yield and quality, but there are few reports on the physiological and biochemical basis for greater yield and quality among virus-free sugarcane seedlings. In particular, the effects of virus-free seedlings on the activity of photosynthetic enzymes and the expression of related genes have not been reported. In addition, results showing increased yield and quality among virus-free seedlings are for sugar cane, and there are only a limited number of studies with chewing cane. Our objective was to systematically evaluate the effect of starting with virus-free chewing cane seedlings on yield and quality, and to explore the physiological and molecular mechanisms behind any increases in yield and quality.

## Results

### Effect of virus-free chewing cane seedlings on yield and quality

The yield of the virus-free seedling No. 7 increased by 29.93% compared with that of infected seedling, and the stem length, stem diameter, internode length, and single stem weight increased by 28.66 cm, 0.52 cm, 2.16 cm, and 27.68%, respectively, all significantly greater than the No. 7 infected seedling (Table 1). The yield of virus-free seedling No. 8 was 21.81% greater than the infected No. 8 seedling, and the stem length, stem diameter, internode length, and single stem weight of the No. 8 virus-free seedling were 34.49 cm, 0.50 cm, 2.68 cm, and 20.10% greater, all significantly greater than the No. 8 infected seedling.

**Table 1:** Effects of virus-free seedlings of chewing cane (*Saccharum officinarum* L.) on yield and quality.

Treatment	Cane yield and composition						Quality parameter			
	Stem length (cm)	Stem diameter (cm)	Internode length (cm)	Single stem weight (kg)	Cane yield per barrel (kg)	Number of effective stems per barrel (strip)	Reducing sugar content (%)	Fiber content (%)	Brix (%)	Sucrose content (%)
VF7	267.88 ± 3.96a^a^	3.23 ± 0.05a	8.94 ± 0.09a	2.26 ± 0.17a	11.20 ± 0.65a	5.5 ± 0.26a	1.74 ± 0.06a	8.15 ± 0.69a	12.21 ± 0.41ab	4.81 ± 0.42ab
VI7	239.22 ± 5.13b	2.71 ± 0.09b	6.78 ± 0.57b	1.77 ± 0.06b	8.62 ± 0.14b	6.2 ± 0.5a	0.83 ± 0.04b	8.08 ± 0.97a	12.26 ± 0.23ab	4.87 ± 0.23ab
VF8	278.99 ± 10.89a	3.15 ± 0.14a	8.79 ± 0.57a	2.39 ± 0.1ab	10.39 ± 0.02a	5.6 ± 0.3a	1.49 ± 0.07a	8.34 ± 0.84a	12.65 ± 0.29a	5.27 ± 0.03a
VI8	244.50 ± 6.17b	2.65 ± 0.23b	6.11 ± 0.42b	1.99 ± 0.06b	8.53 ± 0.28b	5.4 ± 0.6a	0.34 ± 0.06c	10.13 ± 1.22a	11.36 ± 0.03b	3.94 ± 0.19b

VF7, Virus-free seedling No. 7; VI7, Infected seedling No. 7; VF8, Virus-free seedling No. 8; VI7, Infected seedling No. 7.

^a^Different lowercase letters represent significant differences at the level of 0.05.

For the No. 7 cultivar, the sucrose and brix contents decreased by 0.06% and 0.05% (absolute value) compared with the infected seedlings, but the difference was not significant. The reducing sugar content increased by 0.91% (absolute value) in the virus-free seedling, significantly higher than the infected seedling. Furthermore, the fiber content increased by 0.07% in the virus-free seedling compared with the infected, but this difference was not significant.

For the No. 8 seedlings, the sucrose content, brix content, and reducing sugar content were all significantly greater in the virus-free seedlings than the infected, by 1.33%, 1.29%, and 1.15% respectively. The fiber content was not significantly different between the virus-free and infected material.

### Effects of virus-free chewing cane seedlings on chlorophyll content and photosynthetic parameters in leaves

**Figure 1:**
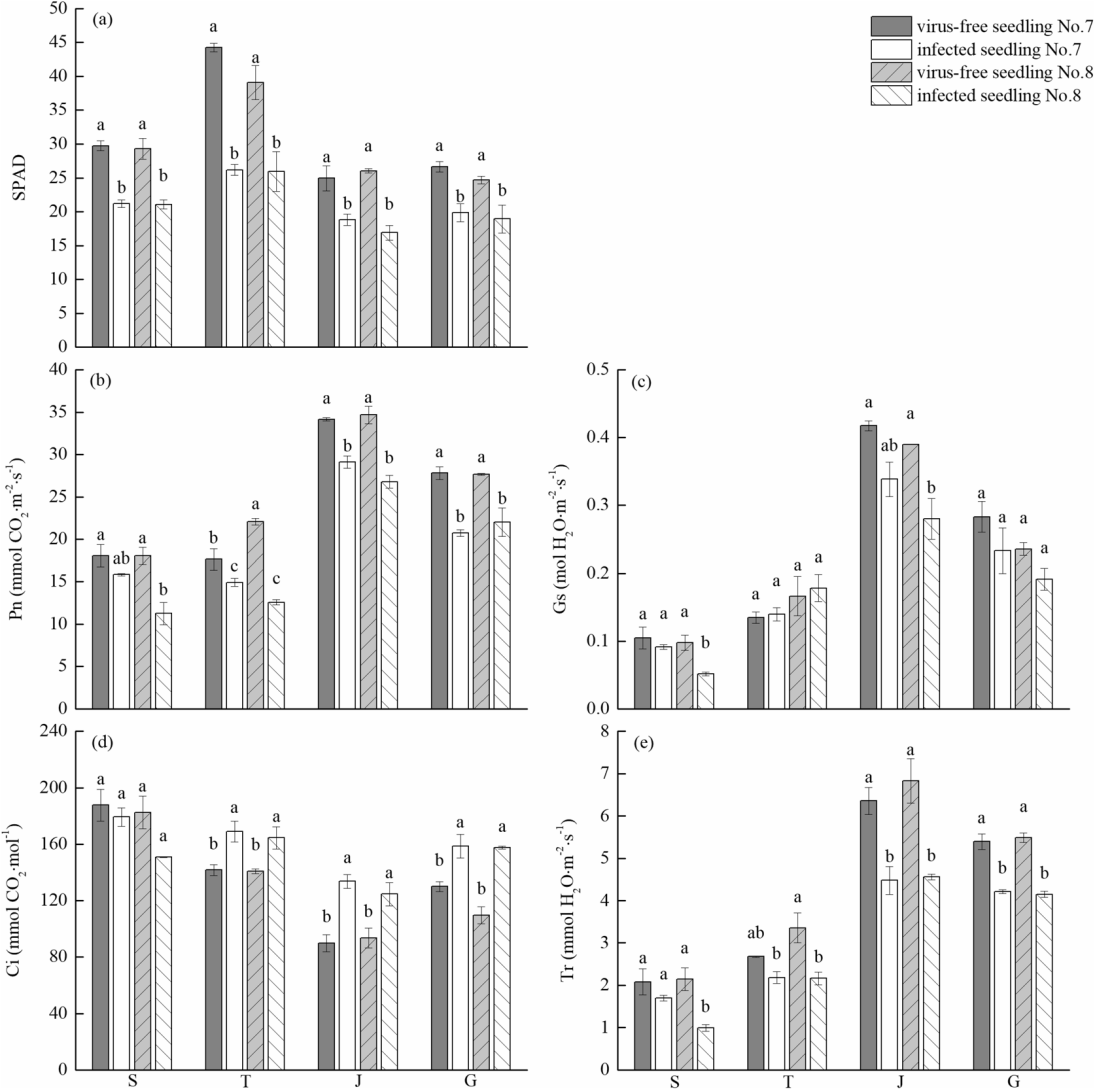
Effects of virus-free seedlings of chewing cane (*Saccharum officinarum* L.) on chlorophyll content (**a**), net photosynthetic rate (**b**), stomatal conductance (**c**), intercellular CO_2_ concentration (**d**) and transpiration rate (**e**). Different lowercase letters indicate significant differences between the virus-free and infected seedlings of the same growth period (*p* < 0.05). S: seedling stage; T: tillering stage; J: jointing stage; G: large growth stage.

With the continuation of the growth period, chlorophyll content initially increased but then decreased (Fig. 1a). The maximum chlorophyll content was measured at the tillering stage, after which it decreased rapidly, and the change in chlorophyll content between virus-free and infected seedlings was the same. The chlorophyll content of the virus-free seedlings was significantly higher than that of the infected seedlings at all growth stages (Fig. 1a).

With the development of growth period, the net photosynthetic rate of leaves initially increased and then decreased, reaching the maximum value at the jointing stage (Fig. 1b). The change in the photosynthetic rate of virus-free and infected seedlings mirrored each other. Except for the seedling stage, the net photosynthetic rate of virus-free seedling No. 7 was not significantly greater than the No. 7 infected seedling, and the net photosynthetic rate of the two virus-free seedling cultivars were significantly greater their infected counterparts in the other growth stages.

The stomatal conductance of leaves initially increased first over time, and then decreased, reaching the maximum at jointing stage (Fig. 1c). The stomatal conductance of virus-free seedlings and infected seedlings had the same trend. The stomatal conductance of virus-free seedlings at the seedling stage, jointing stage, and large growth stage was higher than that of their infected seedlings, but only the stomatal conductance of virus-free seedling No. 8 at seedling stage and jointing stage was significantly higher than that of its infected seedling.

The intercellular CO_2_ concentration of leaves decreased first and then increased, reaching the maximum at seedling stage (Fig. 1d). After which, the CO_2_ concentration decreased and then rose again after the jointing stage. The trend of the intercellular CO_2_ concentration of virus-free seedlings and infected seedlings was the same. There was not a significant difference in the intercellular CO_2_ concentration between virus-free and infected seedlings at the seedling stage, but the intercellular CO_2_ concentration of virus-free seedlings was significantly lower than the infected seedlings at the tillering stage, jointing stage, and large growth stage.

The transpiration rate of leaves initially increased and then decreased, reaching the maximum at jointing stage (Fig. 1e). The trend of virus-free seedlings and infected seedlings was the same. The leaf transpiration rate of virus-free seedling No. 7 at the tillering stage was not significantly higher than that of its infected seedling, but at other growth stages, the leaf transpiration rate of virus-free seedlings was significantly higher than the infected seedlings.

### Effects of virus-free chewing cane seedlings on the activity of photosynthetic enzymes PEPC and rubisco

**Figure 2:**
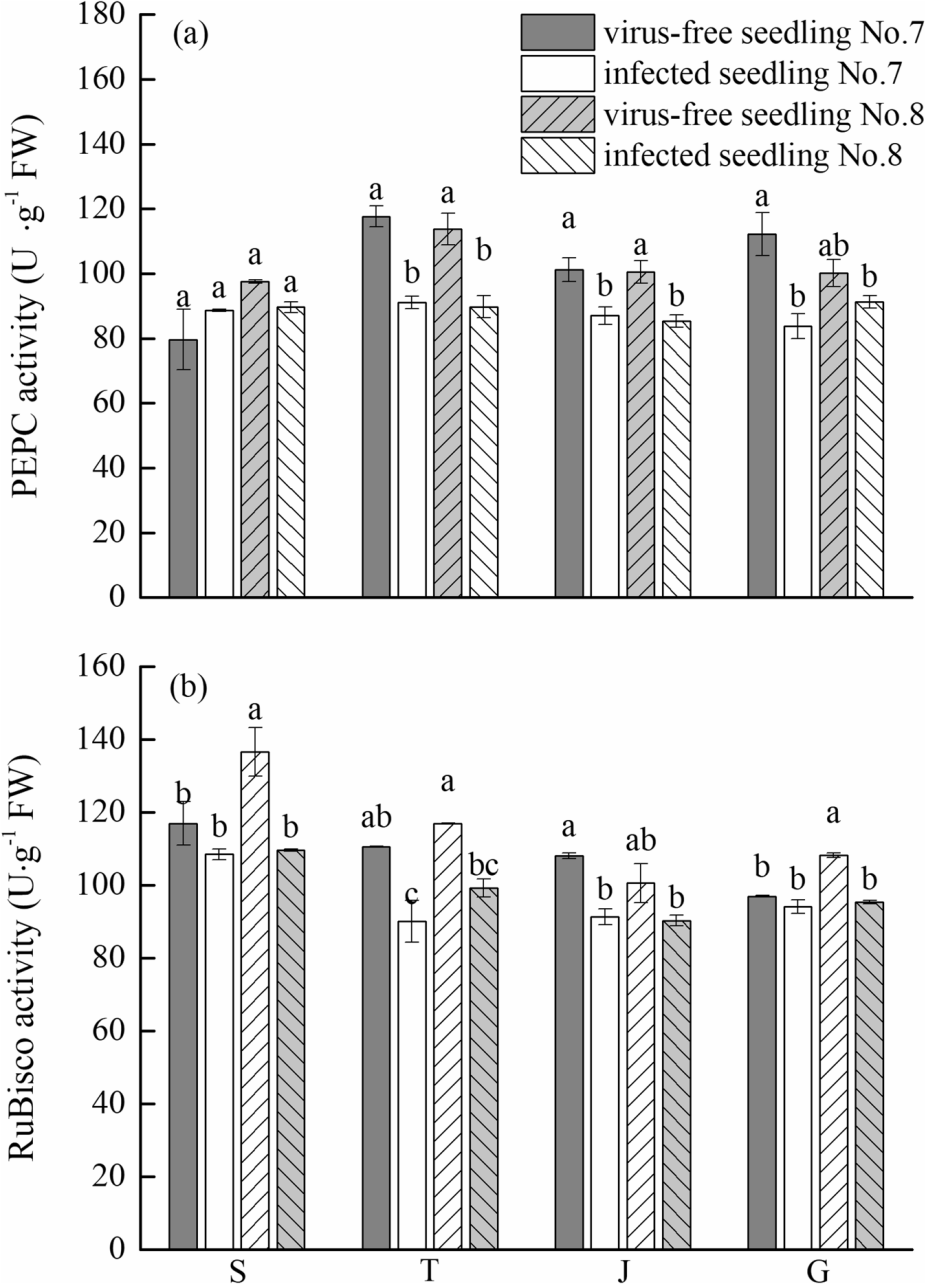
Effects of virus-free chewing cane (*Saccharum officinarum* L.) seedlings on the activity of PEPC (**a**) and activity of Rubisco (**b**). Different lowercase letters represent significant differences at the level of 0.05. S: seedling stage; T: tillering stage; J: jointing stage; G: large growth stage.

With the development of growth period, the activity of PEPC in leaves did not change (Fig. 2a). There were no significant differences in PEPC activity between the virus-free and infected seedlings at the seedling stage, but the PEPC activity of virus-free seedlings at other growth stages was significantly higher than the infected seedlings (except for virus-free seedling No. 8, which did not increase significantly at the large growth stage).

With the development of growth period, the Rubisco activity of leaves had a slow downward trend, and the change trend of virus-free and infected seedlings was nearly the same (Fig. 2b). The Rubisco activity in the leaves of virus-free seedlings was greater than infected seedlings at four growth stages, but the Rubisco activity in the leaves of virus-free seedling No. 7 at the seedling stage and large growth stage, and virus-free seedling No. 8 at jointing stage, did not reach a significant level, compared with infected seedlings.

### Effects of virus-free seedlings of chewing cane on expression of photosynthesis-related genes

**Figure 3:**
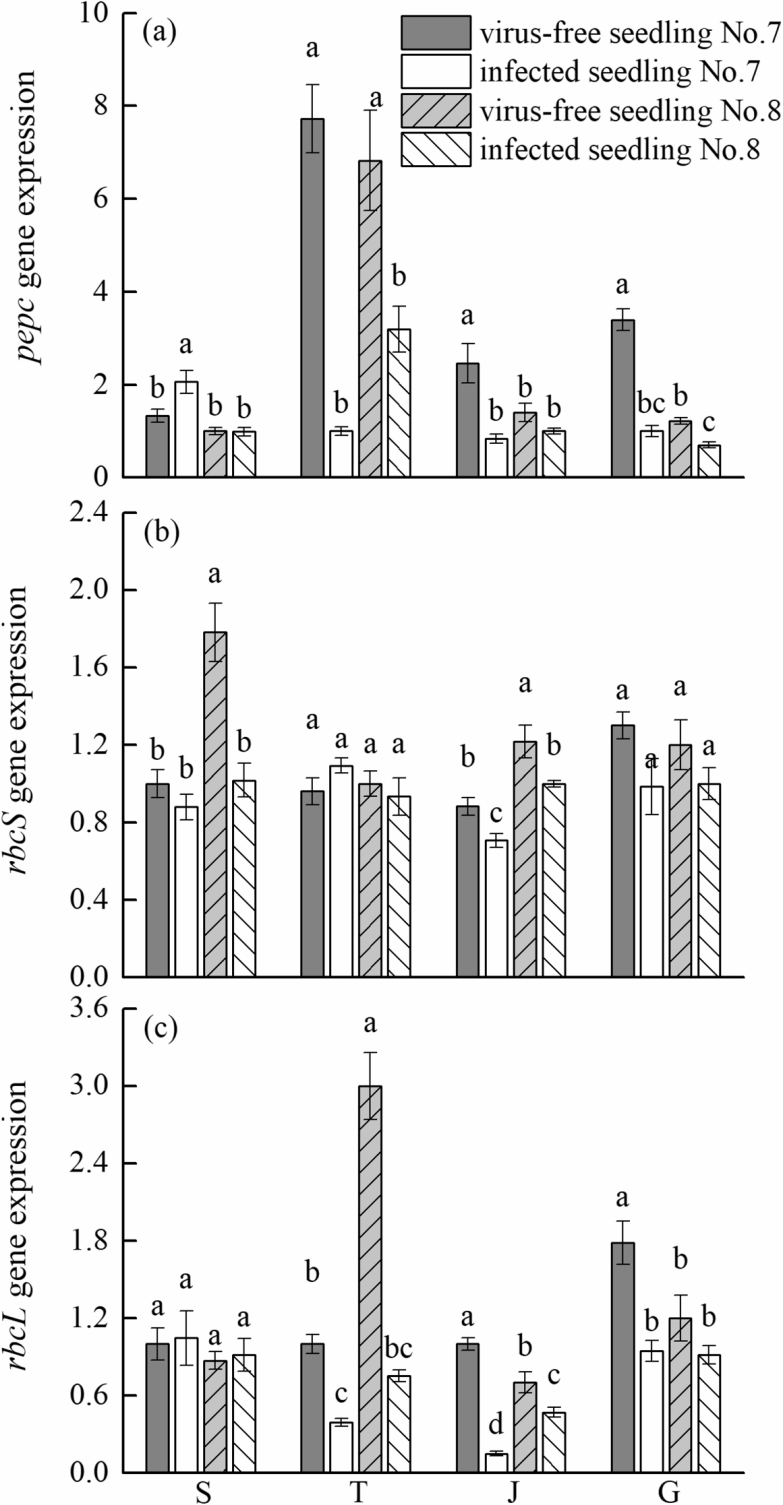
Effects of virus-free chewing cane (*Saccharum officinarum* L.) seedlings on the quantitative expression of *pepc* gene (**a**), *rbcS* gene (**b**), and *rbcL* gene (**c**) in leaves. Different lowercase letters represent significant differences at the level of 0.05. S: seedling stage; T: tillering stage; J: jointing stage; G: large growth stage.

The expression of the *pepc* gene was detected in both virus-free and infected seedlings at four growth stages (Fig. 3a). The highest expression level was found in virus-free seedling No. 7 at the tillering stage, followed by virus-free seedling No. 8 at the tillering stage. The lowest expression level was found in infected seedling No. 8 at large growth stage. There was no significant difference in the *pepc* gene expression level between virus-free and infected seedlings at the seedling stage. The expression level of the *pepc* gene in virus-free seedlings was significantly greater than infected seedlings at the other three growth stages (except that the gene expression level of virus-free seedling No. 8 at jointing stage, which was not significantly increased).

The expression of the *rbcS* gene was detected in both virus-free seedlings and infected seedlings at different growth stages (Fig. 3b). The greatest expression level was in the virus-free seedling No. 8 at the seedling stage, followed by the virus-free seedling No. 8 at the jointing stage. The lowest expression level was in the virus-infected seedling No. 7 at the jointing stage. The expression level of *rbcS* in the virus-free seedling at the seedling stage, the jointing stage, and the large growth stage was greater than the infected seedlings. The gene expression level of virus-free seedling No. 7 and No. 8 at the seedling stage and joining stage (No. 8 only) was significantly greater than the infected seedling counterparts.

The expression of the *rbcL* gene was detected in both virus-free and infected seedlings at four growth stages (Fig. 3c). The highest expression level was found in virus-free seedling No. 8 at the tillering stage, followed by virus-free seedling No. 7 at the large growth stage. The lowest expression level was found in infected seedling No. 7 at the jointing stage. The gene expression level in virus-free seedlings was significantly higher than infected seedlings at the tillering stage, the jointing stage, and the large growth stage (except for virus-free seedling No. 8 at the large growth stage).

### Effects of virus-free seedlings of chewing cane on active oxygen metabolism

**Figure 4:**
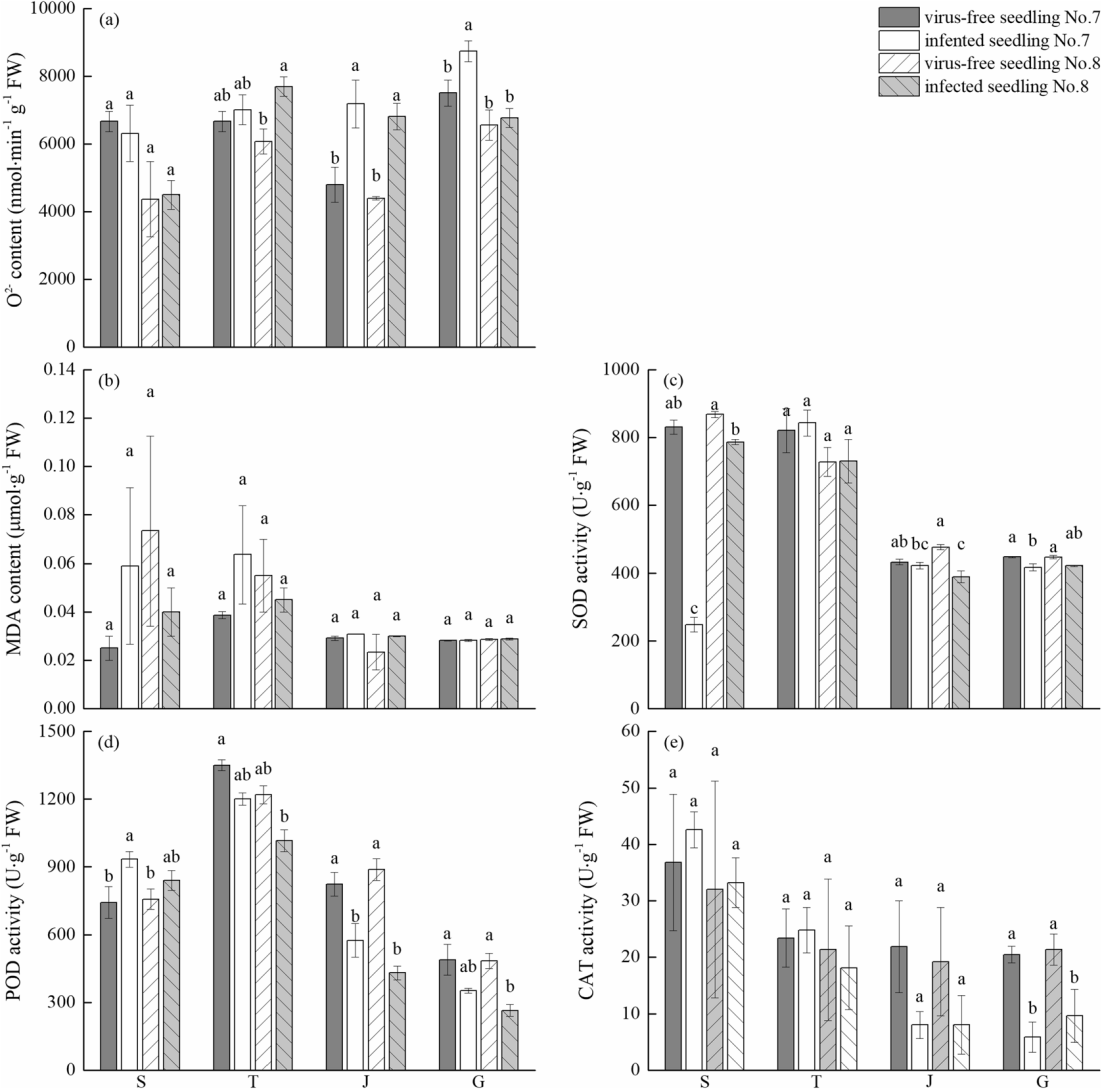
Effects of virus-free chewing cane (*Saccharum officinarum* L.) seedlings on O_2_^-^ content (**a**), MDA content (**b**), SOD activity (**c**), POD activity (**d**), and CAT activity (**e**) of leaves. Different lowercase letters represent significant differences at the level of 0.05. S: seedling stage; T: tillering stage; J: jointing stage; G: large growth stage.

The O_2_^−^ content of leaves of both virus-free and infected seedling No. 7 initially decreased and then increased, reaching the maximum at large growth stage (Fig. 4a). The O_2_^−^ content in leaves of virus-free and infected seedling No. 8 had a similar pattern, but decreased after reaching their maximum at the tillering stage. The leaf content of O_2_^−^ in virus-free seedlings was significantly lower than infected seedlings at the tillering stage, the jointing stage, and the large growth stage (except for virus-free seedling No. 7 at the tillering stage and virus-free seedling No. 8 at the large growth stage).

MDA in the leaves of virus-free seedlings increased first and then decreased, reaching maximum content at the tillering stage (Fig. 4b). MDA in leaves of infected seedlings reached the maximum at seedling stage and then gradually decreased. The MDA content of the leaves of virus-free seedlings was lower than infected seedlings, and there was no significant difference between virus-free seedlings and infected seedling during the four growth stages.

Superoxide dismutase (SOD) activity in leaves of virus-free seedling No. 7, virus-free seedling No. 8, and infected seedling No. 8 reached the maximum activity at the seedling stage and then decreased gradually (Fig. 4c). SOD activity of infected seedling No. 7 increased first and then decreased, and reached the maximum at the tillering stage. At the tillering stage, the SOD activity in the leaves of virus-free seedlings was not different from the infected seedlings. In the remaining three growth stages, the SOD activity in the leaves of virus-free seedlings was significantly greater than infected seedlings (except for virus-free seedling No. 7 at the jointing stage and virus-free seedling No. 8 at the large growth stage).

POD activity of leaves increased first and then decreased, reaching the maximum activity at the tillering stage (Fig. 4d). The change trend of virus-free seedlings and infected seedlings was the same. At the seedling stage, the POD activity of virus-free seedlings was lower than infected seedlings, but the POD activity of virus-free seedlings was greater at the tillering stage, jointing stage, and large growth stage. The POD activity of virus-free seedling No. 7 at the jointing stage and virus-free seedling No. 8 at the jointing stage and the large growth stage increased significantly.

The CAT activity in the leaves of virus-free and infected seedling No. 7 decreased gradually (Fig. 4e). Meanwhile, CAT activity in the leaves of virus-free and infected seedling No. 8 initially decreased and then increased slightly after the jointing stage. The CAT activity in the leaves of virus-free seedlings was greater than that of the infected seedlings at the tillering stage, the jointing stage, and the large growth stage (except virus-free seedling No. 7 at the tillering stage).

## Discussion

Sugarcane is an asexually propagated crop, which is vulnerable to various sugarcane viruses and result in the decline of sugarcane yield and poor quality^13^. Virus-free sugarcane seedling is an effective way to control sugarcane virus disease. It has been reported that virus-free sugarcane seedlings greater yield, but as much as 20%, and that the sucrose content can also be increased^11^. In this study, we compared virus-free and infected seedlings of chewing cane. Stem yield increased by 21.81–29.93%, stem length by 28.66–34.49 cm, internode length by 2.16–2.68 cm, single stem weight by 20.10–27.68%, reducing sugar by 0.91–1.15 (absolute value), and sucrose content by − 0.06–1.33 (absolute value). In this study, we recorded a greater increase in yield in chewing cane due to virus-free seedlings than has been shown in sugar cane. This may be due to the fact that chewing cane has poor stress resistance and is more vulnerable to viral infection than sugarcane^45^. It has been shown that this is particularly the case when it comes to specially the co-infection, where two or more kinds of viruses inhabit chewing cane and result in a significant decline in production^1^. Sugar cane is a hybrid progeny of *S. officinarum* and some wild species, and thus, its resistance to viruses is better than chewing cane^14^. Therefore, we believe that sugar cane may be less likely to be infected by certain viruses than chewing cane, the potential for increasing yield of virus-free seedlings of chewing cane is better than sugar cane.

Photosynthesis is the basis of plant growth and development, and the accumulation of photosynthate is the result of the interaction of Pn, Gs, and Ci. Tr can reflect the intensity of crop transpiration and it is the main driving force for crops to absorb and transport water and mineral elements. The photosynthetic and transpiration rates comprehensively reflect the intensity of plant vitality^15^. Studies have shown that when viruses infect plants, they can cause organelle lesions, reduce the number of chloroplasts in cells, destroy the structure of chloroplasts, increase the activity of chlorophyll decomposition enzymes, and decrease the content of chlorophyll and photosynthetic rate^15–17^. After virus-free treatment, plants can increase leaf chlorophyll content and the net photosynthetic rate, enhancing photosynthesis^18,37^. When infected sugarcane is detoxified, the chlorophyll content and photosynthetic rate of virus-free sugarcane seedlings has increased, and the vitality of virus-free sugarcane plants also increased^19,20^. In this study, the chlorophyll content, and the Pn, Gs and Tr content of virus-free seedlings of chewing cane were higher than those of infected seedlings in each growth stage, and Ci was significantly lower than in infected seedlings. This indicates that after chewing cane seedlings are treated with virus-free treatment, the chlorophyll content, photosynthetic rate, transpiration rate, and the photosynthetic capacity increased. This finding is consistent with previous studies^20–22^. The increase of chlorophyll content and photosynthesis in virus-free seedlings of chewing cane is the basis of increasing chewing cane yield.

PEPC is a key enzyme involved in photosynthesis and has many physiological functions. It is responsible for fixing inorganic carbon in photosynthesis in C_4_ plants to improve the efficiency of photosynthesis and water use^23,24^. When plants are infected by pathogens, the activity of PEPC decreases, limiting photosynthesis and affecting plant growth^25^. Zhang et al.^37^ showed that compared with healthy plants, PEPC activity in sugarcane inoculated with *Leifsonia xyli* subsp. *Xyli* (Lxx), causing sugarcane ratoon stunting disease, decreased significantly, and that photosynthesis was reduced and growth was affected. In this study, PEPC activity of virus-free seedlings of chewing cane was significantly higher than infected seedlings during the tillering stage, jointing stage, and large growth stage. Our findings are similar to Zhang et al.^37^. We did not find a significant difference in the expression level of *pepc* gene between virus-free and infected seedlings at the seedling stage, but the expression level of *pepc* at the tillering stage, jointing stage, and the large growth stage was significantly higher in the virus-free seedlings than the infected seedings. Furthermore, the trend of expression of the *pepc* gene was consistent with the change trend of PEPC activity. We conclude that in chewing cane, the enhanced activity of PEPC in the virus-free seedlings is related to the up-regulation of the *pepc* gene transcription level.

Rubisco is a key enzyme for carbon assimilation in plant photosynthesis, which regulates CO_2_ fixation and photorespiration during photosynthesis. It has been found that Rubisco is composed of large subunits (*rbcL*) and small subunits (*rbcS*)^26^ and that under stress such as pathogen infection, the subunits are down-regulated and Rubisco activity is significantly decreased, affecting plant photosynthesis^27^. Sampol et al.^28^ showed that compared with virus-free plants, viral infection caused a significant decrease in Rubisco activity and photosynthesis. Ji et al.^29^ reported that under phytoplasma infection, the expression of the *rbcL* gene in mulberry leaves was down-regulated, and that Rubisco activity and the photosynthetic rate were significantly reduced. In this study, the activity of Rubisco in the leaves of virus-free seedlings was significantly higher than infected seedlings, and the expression levels of *rbcL* and *rbcS* genes in virus-free seedlings were greater than infected seedlings. We also found that the expression levels of *rbcL* at the tillering, jointing, and large growth stages, as well as the expression levels of *rbcS* at the seedlings and jointing stages, were significantly different from infected seedlings. In this study, the activity of Rubisco in virus-free seedlings was significantly greater than infected seedlings, which may be related to the up-regulation of *rbcL* and *rbcS* gene expression in the virus-free seedlings. The up-regulation of the transcription level of photosynthetic genes and the enhancement of the activity of key photosynthetic enzymes are the photosynthetic physiology and molecular basis for increasing yield and quality of chewing cane virus-free seedlings.

Under normal conditions, the production of reactive oxygen species (O_2_^−^ and MDA) in plant cells is in a dynamic balance with the removal of antioxidant enzymes (CAT, SOD, and POD) and does not cause damage to plants^30^. However, under stress, this balance is destroyed, resulting in the accumulation of reactive oxygen species, which directly or indirectly induce membrane lipid peroxidation^31^. Related studies have shown that plants can improve antioxidant enzyme activity, reduce free radical (O_2_^−^) content, and increase resistance to stress by virus-free treatment^15^. Kang^32^ showed that MDA content in virus-free potato seedlings was lower than common potato seedlings, and that the activities of protective enzymes such as CAT, SOD, and POD were significantly increased. Thus, this effectively prevented the damage of cell membrane structure by membrane lipid peroxidation. In this study, the antioxidant enzymes activities of SOD, POD, and CAT in leaves of virus-free chewing cane seedlings were higher than infected seedlings, and the content of O_2_^−^ and MDA in leaves of virus-free chewing cane seedlings was lower than infected seedlings at all main growth stages. This indicates that the virus-free seedlings of chewing cane have a greater antioxidant capacity, have less membrane lipid peroxidation, and a greater stress resistance. In addition, previous studies^33,34^ show that under the stress of pathogens, sugarcane plants accumulate reactive oxygen species, destroy the redox system, and decrease antioxidant enzymes activities of POD, SOD, and CAT ,which also verified the results of our study from another perspective.

## Conclusion

The chlorophyll content, the activity of photosynthetic key enzymes, the expression levels of photosynthetic key genes, and photosynthesis of chewing cane were all enhanced in virus-free seedlings. In the virus-free seedlings, the content of O_2_^−^ and MDA of virus-free seedlings decreased, the activities of antioxidant enzymes SOD, POD, and CAT of increased, and the stress resistance increased. Virus-free seedlings had a significant effect yield and quality. Cane yield increased by 21.81–29.93%, stem length increased by 28.66–34.49 cm, internode length increased by 2.16–2.68 cm, single stem weight increased by 20.10–27.68%, reducing sugar increased by 0.91–1.15% (absolute value), and sucrose content increased by − 0.06–1.33% (absolute value).

## Materials and methods

### Plant materials

Virus-free seedlings and infected seedlings of the Chinese chewing cane cultivars No. 7 and No. 8 were used. These genotypes were bred from the natural variant plants of ‘Badila’, the primary Chinese chewing cane cultivar, using the systematic selection method. Chewing cane plants without disease symptoms and with obvious symptoms of sugarcane mosaic disease were selected from the secondary virus-free seedling nursery at the sugarcane breeding base of South China Agricultural University. Molecular methods were used to detect major viruses and diseases, including ratoon stunting disease^2,35^. Plants with obvious symptoms of sugarcane mosaic disease were co-infected by SrMV and SCMV (Figure S1a,b), and the plants without disease symptoms were negative (Figure S1c,d).

### Experimental conditions

The detected healthy virus-free and co-infected chewing cane stalks were selected cut into one-budded setts, and the full and fresh setts were selected as planting materials. The experiment was carried out in the insect-free greenhouse at the sugarcane breeding program of South China Agricultural University in Guangzhou, China. On February 4th, 2018, 5–6 one-bud setts were planted per barrel (35.0 cm high, 38.5 cm diameter at top, and 34.0 cm diameter at bottom). Thirty kg of sugarcane field soil were used in each barrel, and the rates of available nitrogen, available phosphorous and available potassium were analyzed before planting, which were 72.62, 50.17 and 52.42 mg/kg, respectively. At the three-leaf stage, 4–5 seedlings of a similar size were retained in each barrel. Nine barrels of virus-free seedlings and nine barrels of infected seedlings were planted for both No. 7 and No. 8 cultivars. The experimental design was a single factor contrast test with three repetition. Thus, three barrels were one replicate. Planting management refers to the normal production and management of chewing cane^36^. The heart leaves of plants were collected at seedling stage, tillering stage, jointing stage, and large growth stage^37^, and stored in refrigerator at -80℃ for later use to determine the physiological indexes and gene expression level. The experiment was harvested on November 28, 2018.

### Investigation of agronomic traits and determination of yield and quality

At the time of harvest, stem length, stem diameter, internode length, single stem weight, and stem yield per barrel were measured using similar methods as outlined by Zhang et al.^37^. At the maturity stage, 6 normal chewing cane stems (4 main stems and 2 tillers stems) were selected for each treatment (virus-free seedlings No. 7 and No. 8, infected seedlings No. 7 and No. 8), and brix, sucrose content, reducing sugar, and fiber content were measured using the methods outlined by Li and Zheng^38^.

### Determination of chlorophyll content and photosynthetic parameters in leaves

Chlorophyll content and the photosynthetic parameters in leaves at the seedling stage, tillering stage, jointing stage, and large growth stage were determined. Chlorophyll content, net photosynthetic rate (Pn), stomatal conductance (Gs), intercellular CO_2_ (Ci), and transpiration rate (Tr) of the + 1 leaves of plants were determined using a chlorophyll meter (SPAD-502 plus, Konica Minolta Holdings, Inc., Tokyo, Japan) and portable photosynthetic apparatus LI-6400XT (LI-COR, USA) from 9:00 to 11:00 am.

### Determination of key enzyme activities in photosynthesis

The activity of phosphoenolpyruvate carboxylase (PEPC) and ribulose-1,5-bisphosphate carboxylase (Rubisco) within leaves were determined using the ELISA kit (Shanghai Enzyme Biotechnology Co., Ltd., China) according to the manufacturer's instructions.

### Quantitative expression of key photosynthetic genes

Total RNA was extracted from leaves using the TRlzon total RNA extraction kit (Vazyme Biotech Co., Ltd., Nanjing, China) according to the manufacturer's instructions. The total RNA concentration and purity were determined using the ultra-micro spectrophotometer (NanoDrop 2000C, Thermo Scientific,Wilmington, DC, USA). The 1 μg of RNA was reverse-transcribed into cDNA using the HISCRIPT RT SuperMix for qPCR (+ g DNA wiper) kit (Vazyme Biotech Co., Ltd., Nanjing, China) and stored at  − 20 °C for further use.

According to the sequence of the sugarcane *pepc* gene (GenBank Acc. No. AY135709), the small subunit gene *rbcs* of Rubisco (GenBank Acc. No. JN591757) and the large subunit gene *rbcL* of Rubisco (GenBank Acc. No. AE009947), the fluorescent quantitative specific primers of *pepc*, *rbcs,* and *rbcL* were designed using Primer 5.0 software. Primers with good linearity, high amplification efficiency, and specificity were screened for subsequent experiments (Table 2). The *GAPDH* gene of sugarcane was used as internal control gene^39^.

**Table 2:** Fluorescent quantitative primer sequences for *pepc*, *rbcS*, *rbcL,* and *GAPDH* genes.

Gene	Primer sequences
*pepc*	F: 5′-AAACTGTATGGAAGGGTGTG-3′
	R: 5′-CAAGCATACGTCTCTTGTCA-3′
*rbcS*	F: 5′-GCAAGGAAGGCTTCGTGTAC-3′
	R: 5′-GTCTGCCTGATGTTGTCGAAG-3′
*rbcL*	F: 5′-TCTACGCGGTGGACTTGATT-3′
	R: 5′-AATGCCCCTTGATTTCACCG-3′
*GAPDH*	F: 5′-CACGGCCACTGGAAGCA-3′
	R: 5′-TCCTCAGGGTTCCTGATGCC-3′

The reaction was performed using the CHAMQ Universal SYBR qPCR Master Mix kit (Vazyme Biotech Co., Ltd., Nanjing, China). The experiment was carried by using cDNA as a template, preparing a fluorescence quantitative reaction system, and setting a reaction procedure according to the instructions. The amplification reaction was performed on a real-time fluorescent quantitative PCR instrument (CFX96, Bio-Rad, Hercules, CA, USA). The expression level of the gene relative to the internal control gene *GAPDH* was analyzed using the 2^−ΔΔCt^ method^40^, and three replicates were performed on each sample.

### Determination of malondialdehyde (MDA) content and superoxide anion (O_2_^-^) content in leaves

MDA content in leaves was determined based on the methods outlined by Zeng et al.^41^, and superoxide anion (O_2_^−^) content was determined using methods described by Wang and Luo^42^.

### Determination of defense enzyme activity in leaves

The crude enzyme solution was extracted according to Moerschbacher et al.^43^ with slight modification. 1 g leaf sample was weighed and ground with liquid nitrogen, and then added to pre-cooled 2 mL 0.1 mol/L pH 8.8 boric acid buffer (containing 1 mmol/L EDTA-Na_2_ and 1% PVPP). The leaf was quickly ground. The homogenate was then transferred into a 10 mL centrifugal tube, and then the extract of 2 mL was taken to collect the homogenate adhering to the mortar and the mortar rod. After collection, the homogenate was transferred into the centrifugal tube and the volume was fixed to 10 mL with a buffer solution. The sample was centrifuged for 15 min (4 °C, 12,000 r/min) and the supernatant was collected and stored in a refrigerator at  − 20 °C for further enzymatic activity determination. The SOD activity was determined using the method outlined by Beauchamp and Fridovich^44^. The peroxidase (POD) activity was determined according to Xu et al.^45^, and the catalase (CAT) activity determination method is described by Aebi^46^.

### Statistical analysis

The same index for each treatment were measured three times, and the data statistics were performed in Excel 2010 (Microsoft Corporation, Wilmington, DC, USA). One-way ANOVA with Duncan's new multiple range test was used to analyze the significance of the differences between each group using IBM SPSS 19.0 statistical software (IBM, New York, USA). Means were thought to be significantly different and marked with lowercase letters (i.e. a,b), when *p* < 0.05. The figure draw using Origin 9.0 software (OriginLab, Massachusetts, USA).

## Supplementary information

Supplementary information
