# Supplementary material for: Physiological and molecular mechanisms governing the effect of virus-free chewing cane seedlings on yield and quality
Source: Sci Rep. 2020 Jun 25;10:10306. doi: 10.1038/s41598-020-67344-4 (PMC7316764; doi:10.1038/s41598-020-67344-4)

**Physiological and molecular mechanisms governing the effect of virus-free chewing cane** **seedlings on yield and quality**

Kai‒li Wang ^1, 2 #^, Quan‒qing Deng ^1, 2 #^, Jian‒wen Chen ^1, 2^, Wan‒kuan Shen ^1, 2^ *

**Figure** **S1** Detection of SrMV(a & c) and SCMV (b & d) in plants with obvious symptoms of sugarcane mosaic disease (a & b) and plants without disease symptoms (c & d) of chewing cane cultivars No. 7 and No. 8 by RT-PCR. M: DL 2000 DNA ; +: positive control; ‒: negative control; 1-5: plants of chewing cane cultivar No. 7; 6-10: plants of chewing cane cultivar No. 8.

**Figure S1**


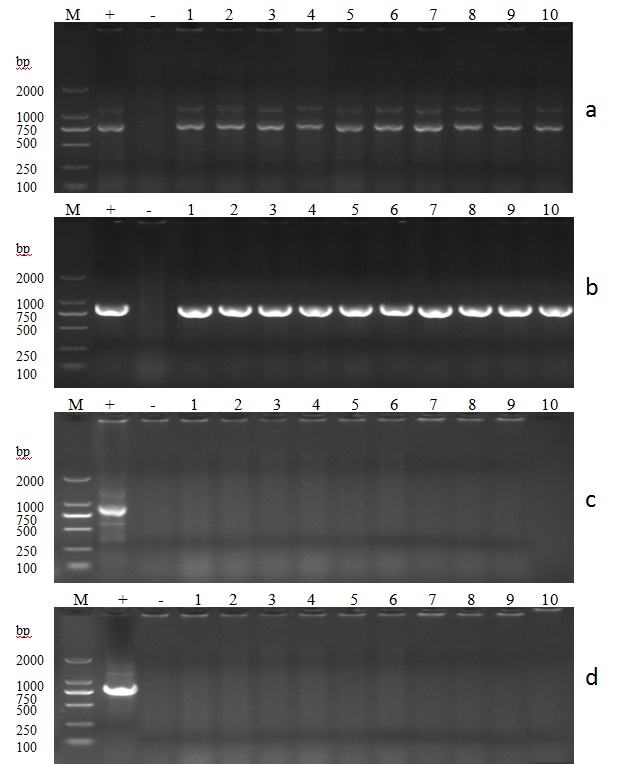

Supplement: Supplementary file 1 — Supplementary information [file 41598_2020_67344_MOESM1_ESM.doc]
